# Pyæmia in the Dog1Read before the Montreal Veterinary Medical Association, November 7, 1895.

**Published:** 1896-02

**Authors:** Harri H. Dell


### PYÆMIA IN THE DOG.[1]

BY HARRI H. DELL.

I HAVE been guided in the selection of my subject for your consideration this evening by a desire to direct your attention to a disease but little referred to in our literature. I was quite unaware of the paucity of information in the text-books concerning pyæmia in the dog until I began to consult the several libraries to which I had access. Later on I will report in detail a case which came under my own observation and following an operation of the most trivial nature.

Pyæmia is a disease characterized by recurrent chills and intermittent fever, with the formation of abscesses in various parts of the body resulting from contamination of the blood by the bacteria of suppuration. The point from which the bacteria and their products are distributed is usually a suppurating wound. Thrombi frequently result, degenerate, and break down, the fragments being carried along in the blood-stream, and thus convey the infection to organs far removed from the original seat of suppuration. The lymphatic system also may convey the infective material to distant organs. The disease is somewhat insidious in nature, and for this reason frequently obtains a foothold upon the animal before it is suspected.

The symptoms which are presented are occasioned by the inflammatory processes and the metastases. Fever of an intermittent type, accompanied by chills, is seen in all cases. The other symptoms vary with the organs most affected, and when we bear in mind the many organs in which the metastases occur, we are not surprised at the complexity of the symptoms.

Both pyæmia and septicæmia usually have their origin in wounds, but cases have been recorded where no wound has been discovered, these apparently idiopathic cases being designated as of spontaneous origin; but, reasoning from analogy rather than with our practical experience with the disease, I think the doctrine of spontaneity no more tenable in this particular disease than in others.

Pyæmia and septicæmia are, I think, the unsuspected cause of much of the mortality following surgical operations in

[1] Read before the Montreal Veterinary Medical Association, November 7, 1895.

veterinary practice. The unfavorable terminations are hastily concluded to be the result of other causes than the entrance into the animal economy of organisms whose potency in the causation of diseases is beyond doubt. Diagnoses are made, but how rarely are autopsies performed to verify the same. To introduce into our practice the conditions of asepsis and antisepsis obtained in our well regulated human hospitals is obviously quite impossible. But do we as practitioners always endeavor to apply as far as possible in our practice the doctrines of Lister? Are we not frequently careless in this respect, and because we cannot easily attain perfect conditions do we not satisfy ourselves with a minimum amount of effort in that direction, and then blindly attribute failure to anything rather than our own carelessness or neglect?

Our English text-books on the practice of medicine and surgery contain descriptions of pyæmia based for the most part on the experiments of Koch, and refer to the occurrence of the disease in horses, cattle, and sheep, but singularly enough no cases are recorded of the disease in dogs.

Our cynological literature also treats but little of the disease. Woodruff Hill is the only one of the many writers I have consulted who refers to the disease. He advises strict cleanliness in the treatment of even small wounds, as "pyæmia and septicæmia may follow carelessness in this respect."

The French works I have had at my disposal give the matter very little notice. Rolle's *Pathologie Morbide* contains a good account of the disease, but makes no reference to its occurrence in the canidæ, and more surprising is the fact that in the *Maladies des Chiens* (a French translation of Hertwig), I failed to find either pyæmia or septicæmia mentioned among the lists of diseases to which the dog is liable. In the *Deutsche Zeitschrift für Thier medicin*, vol. ix., p. 92, pyæmia is mentioned as occurring in young dogs following omphalitis. In the *Monatsheft für Thier Heilkunde*, vol. lv., p. 381, Frohner records a case of pyæmia in the bitch following an attack of endometritis. Rabé in the *Berliner Thierarzthche Wochenschrift*, 1888, p. 65, describes the formation of multiple abscesses due to an organism resembling actinomyces.

The case referred to in my opening remarks was that of an aged cocker spaniel dog, which was brought to the college hospital on October 4th, with the history of having the end of his tail crushed by being run over by a street car. Amputation was performed at the articulation between the fifth and sixth coccygeal vertebræ. The animal continued in good health, but the local conditions not being satisfactory, it was decided to excise another vertebræ and leave a flap of skin to form the stump. This was done on October 12th. The parts were washed before the operation with soap and water, followed by a 1 per cent. solution of carbolic acid. Hands and instruments were also subjected to similar antiseptic treatment. Excision being affected, the edges of the skin were brought into apposition by silk sutures, the wound dusted with iodoform, and a bandage applied. The wound was dressed on October 14th, and found to be healing by granulation at the upper part, while from the lower there was a very slight discharge of a serous nature.

On the 16th there was no evidence of a discharge, the entire wound appeared to be united sufficiently to remove the sutures. The first symptom of any indisposition that presented itself was anorexia on the morning of the 17th, and then the temperature was found to be 101° F.

At 9 P.M. the thermometer registered 104°, pulse 80, and, as the animal still refused food and appeared to be very weak and depressed, beef-tea and stimulants were administered. On the morning of the 18th the only change in the symptoms was such increased weakness that a fatal issue seemed very near. At 11 A.M. he was carried into the cynology clinic, where he expired before any physical examination could be made.

Dr. Mills, from the history, diagnosed pyæmia or septicæmia, which was verified later by post-mortem examination.

The autopsy was held nine hours after death and revealed the following conditions: Serous cavities dry, intestines distended, serous coats showed reddened patches, but there were no signs of peritonitis.

*Spleen:* Extending along the middle of the abdominal cavity it was greatly enlarged, congested, soft, with numerous large hemorrhagic infarcts and minute abscess formations.

*Kidneys:* Enlarged, capsule easily removed, surfaces smooth, pale and mottled, with areas of congestion and hemorrhages. On section showed great fatty degeneration, large white necrotic infarcts, but no signs of multiple abscesses visible to the naked eye. Pelves and ureters injected.

*Liver* large, congested, fairly firm, without the presence of abscesses. There were also several small superficial areas of necrosis.

The *intestines* contained much mucus and a specimen of tænia 75 cm. in length. Otherwise no evidence of disease.

The *heart* was normal in size, containing a moderate amount of blood, blue-black in color, and also small quantity of mixed clot. The muscle was firm, deep-red in color, and free from abscesses. Aortic valves were chronically thickened at their attached margins. The right posterior valve had recent dark-colored vegetations upon it, while behind it was a small recent thrombus. The left posterior valve was greatly degenerated, its free margin showing much loss of substance and numerous vegetations. At the attached margin were two deep ulceration, involving the heart substance. There was also slight fusion of the valves.

*Lungs:* The bronchi were slightly reddened, larger vessels free; otherwise no evidence of pulmonary disease. The brain was normal.

*Tail:* Extremity presents small sloughy aperture without macroscopic evidence of thrombi.

The articulations showed no traces of abscess formation.

Microscopic examination of organs stained with methylene-blue revealed the following results:

In the *spleen* numerous large hemorrhages and very minute abscesses, and throughout small collections of cocci (apparently staphylococcus pyogenes), evidently of embolic origin.

*Kidneys* showed a similar condition and also advanced parenchymatous degeneration.

In the *liver* were hemorrhages, old and recent, and areas of necrosis. Cells in the centre of the lobule showed early atrophy. No true abscesses were apparent, but cocci were present in arteries and veins and invading the bile ducts.

The *heart* muscle showed cocci in the capillaries, but no abscess formation.

*Tail:* A section from the site of the wound showed considerable necrosis, small cell infiltration, together with granulation tissue. The veins themselves, so far as could be made out, did not show, on being stained with methylene-blue, any trace of bacteria in their lumen. Loose thrombi, and some partially organized, were seen in the vessels, but further examination was rendered unsatisfactory owing to conditions under which the tail had been preserved.

*Conclusions and Pathological Diagnosis.* Death from *pyæmia* following the operation on the tail. Thrombo-phlebitis in the vessels. Acute ulcerative aortic endocarditis with metastatic abscesses in the kidney and spleen capsule, parenchymatous nephritis, necroses in liver.

*Interesting Features.* It is interesting to notice the virulence of the disease following so simple and trivial an operation.

Further, the very few clinical symptoms are also not what would be expected in a disease making such rapid advances, while again the freedom of the lungs and articulations from abscesses is one particular wherein the disease in this case did not follow the usual course as seen in other animals.

It affords me great pleasure to acknowledge the most valuable assistance rendered me in the laboratory work on this case by Drs. Adami and Martin of the pathological laboratory of the University.

